# Autogenous dentin-derived membrane as a biologic alternative to collagen membrane for guided bone regeneration in immediate implant placement in the posterior mandible: a randomized clinical trial

**DOI:** 10.1007/s10266-025-01271-6

**Published:** 2025-12-10

**Authors:** El Hussein Farag Alsayed Mohamad, Sally Awad, Mahy Hassouna Abbas, Heba Abo-Elfetouh Elsheikh

**Affiliations:** 1 Oral and Maxillofacial Surgery Department, Faculty of Dentistry, Mansoura University, Mansoura, Egypt; 2 Fixed Prosthodontics Department, Faculty of Dentistry, Mansoura University, Mansoura, Egypt

**Keywords:** Dental implants, Dentin, Collagen, Membranes, Guided tissue regeneration

## Abstract

In implant dentistry, demineralized dentin matrix is frequently utilized as a bone substitute as it contains many growth factors. This study aimed to evaluate the clinical and radiographic outcomes of an autogenous dentin-derived barrier membrane compared with a collagen membrane for guided bone regeneration in immediate dental implant placement in the mandibular posterior region. A total of 40 implants were placed in this randomized clinical study involving 35 patients who required extraction and replacement of non-restorable mandibular premolar or molar teeth with implants. The patients were randomly divided into two equal groups. In Group I, 20 implants were placed immediately after extraction using a dentin-derived barrier membrane, while in Group II, 20 implants were placed immediately with a collagen membrane. Evaluations were performed immediately postoperatively and after 3 and 9 months to assess implant stability, Landry healing index, modified sulcus bleeding index, peri-implant pocket depth, marginal bone loss, and relative bone density. A total of 20 female patients and 15 male patients were enrolled in this study. No statistically significant differences were found between the two groups regarding implant stability, soft tissue parameters, marginal bone loss, and relative bone density at different time intervals (*P* > 0.05). The dentin-derived barrier membrane appears to be effective for successful guided bone regeneration. Therefore, in implant dentistry, it could be considered a novel, natural collagen membrane and may represent a cost-effective autogenous alternative to commercially available collagen membranes. *Trial registration:* This study was registered under registration number NCT06615362 on www.clinicaltrials.gov on 26/9/2024.

## Introduction

The greatest obstacle in implant dentistry remains the placement of dental implants in areas with insufficient alveolar bone. Due to the natural loss of bone following tooth extraction, bone augmentation is frequently required to achieve the optimal implant position for prosthetic replacement [1]. Alveolar bone loss before tooth extraction may result from periapical pathology, periodontal disease, or trauma to the teeth and surrounding bone. Additionally, bone loss may occur as a result of extraction-related injury [2]. A prospective clinical study demonstrated that the posterior segment of the alveolar ridge lost approximately 33% of its width within the first three months and 50% after one year [3]. Adequate bone width and height are essential for proper prosthetic implant placement. This makes the treatment of patients who have lost teeth in the posterior mandible particularly challenging as bone loss occurs rapidly in this region and is further aggravated by the absence of physiological loading [4].

Immediate implant insertion combined with guided bone regeneration (GBR) has the advantage of minimizing bone loss during the physiological remodeling phase of socket healing [1]. Both procedures have been shown to provide successful and predictable results in the posterior mandible [5].

In GBR procedures, it is essential to eliminate epithelial and connective tissue cells from the area of the defect that is to be regenerated, ensuring that only bone cells can proliferate in the defective region. Therefore, in order to effectively direct the regeneration process, non-resorbable or bioabsorbable membranes are necessary [6].

A barrier membrane should possess the following desirable properties: biocompatibility, cell-occlusive capability, host tissue integration, clinical manageability, space-maintaining ability, and sufficient mechanical and physical strength [7].

A collagen membrane is the most commonly used natural membrane in GBR because it promotes wound healing through cell–matrix interactions and supports bone formation. It exhibits a variety of cell morphologies, possesses antibacterial properties, and is easily obtainable from various species [7].

One of the fundamental principles of GBR is the use of bone grafting material. They serve as internal framework to support the membrane. The biological rationale for the use of filler materials mostly comes from their ability to scaffold, maintain space, and stabilize blood clots [8].

Biocompatible and multifunctional biomaterials with unique chemical, mechanical, and biological properties have been developed as a result of ongoing scientific developments. Because of their capacity to interact with the surrounding tissue environment and elicit certain biological responses, these materials have excellent cyto-compatibility and bioactivity. These characteristics have made bioactive biomaterials essential to tissue repair and regenerative medicine, with a wide range of uses in dentistry and medical domains. They help with orthopedic bone repair in medicine, and they are utilized in dentistry for bone grafting, preimplant bone defect, and periodontal regeneration. This development emphasizes how important it is to choose appropriate biomaterials for GBR [9].

Autogenous bone from the same patient is the gold standard graft material because it is osteogenic, osteoconductive, and osteoinductive. However, autogenous bone harvesting necessitates additional surgical procedures [10].

Both dentin and bone are mineralized tissues with nearly identical chemical compositions [11]. By weight, they consist of approximately 70% hydroxyapatite, 18% collagen, 2% non-collagenous proteins, and 10% body fluids. Dentin is an avascular connective tissue and therefore exhibits low antigenicity [11]. A demineralized dentin matrix (DDM) from human teeth is widely used as a bone substitute in implant dentistry [12]. The composition of DDM is also similar to deproteinized bovine bone mineral (DBBM), which mostly consists of hydroxyapatite. Both are osteoinductive and osteoconductive [13].

In cases of hopeless teeth, dentin—which constitutes the majority of a tooth—can serve as an abundant and innovative resource for medical recycling. It is considered a novel material for GBR [14, 15] and represents a promising option as it is more cost-effective than other conventional methods [11, 14].

To the best of our knowledge, no previous study has evaluated the use of an autogenous dentin-derived barrier membrane (DDBM) specifically in immediate implant placement, nor has any study directly compared it with the collagen membrane, which remains the most extensively used barrier material in GBR procedures. Therefore, the objective of this study was to evaluate and compare the clinical and radiographic outcomes of DDBM and collagen membrane for GBR in immediate dental implant placement in the posterior mandible. This study’s null hypothesis was that there were no significant differences between autogenous DDBM and collagen membrane for GBR of immediate dental implant in posterior mandible regarding implant stability, soft tissue parameters, marginal bone loss (MBL), and relative bone density.

## Materials and methods

The patients in this study were selected from the Outpatient Clinic of the Department of Oral and Maxillofacial Surgery, Faculty of Dentistry, Mansoura University, Mansoura, Egypt. All participants required extraction of non-restorable posterior mandibular teeth and replacement with immediate dental implants. The study protocol was approved by the Ethical Committee of the Faculty of Dentistry, Mansoura University (Approval No. A04010222), in accordance with the seventh revision of the Declaration of Helsinki (2013). Written informed consent was obtained from all participants after they were informed of the potential risks and benefits of the procedure.

### Patients’ selection

The following criteria were used to select the study’s patients:

### Inclusion criteria

1. The age range was between 18 and 45 years.
2. Mandibular premolar or molar tooth/teeth that couldn’t be restored and required extraction and GBR to place an implant immediately after extraction.
3. There were no clinical or radiographic indications of an acute infection, draining fistula, or pus exudate at the surgical site or on the tooth that was scheduled for extraction.
4. At least 5 mm of available bone from the root apex to the superior border of inferior alveolar was present.
5. Adequate inter-arch space for good prosthesis.
6. Cooperative patient without a history of parafunctional habits or bruxism, with a high level of motivation and acceptable oral hygiene.
7. The patients were free of systemic diseases that would make the implant surgery absolutely contraindicated.

### Exclusion criteria

- 1. Smokers.
- 2. Pregnant women.
- 3. The patients who received radiotherapy to the head and neck region.

### Calculating the sample size

The sample size calculation was based on the mean bone density values reported in a previous study by Mizar et al.[16]. Using a two-tailed test with an α error of 0.05, a power of 90%, and an effect size of 1.06, the sample size was determined with G*Power software (version 3.1.9.7). The required total sample size was calculated to be at least 20 implants per group.

### Randomization

For randomization, a computer-generated list was created using SPSS software (version 27.0). The allocation sequence was managed by a senior resident from the department who was blinded to the treatment assignments and did not participate in the clinical procedures. Patients were randomly assigned in a 1:1 ratio to two groups, each comprising 20 implants.

### Blinding

The operator was not involved in patient allocation or outcome assessment. Although operator blinding was not feasible due to the nature of the surgical procedure, none of the patients were aware of their group assignment. The treatment allocation was concealed from the independent assessor who performed the outcome evaluations. Additionally, the statistician responsible for data analysis was blinded to the group assignments.

### Study design

This prospective randomized clinical trial was conducted in accordance with the CONSORT principles for conducting clinical studies (CONSORT Flowchart). Two equal groups were assigned randomly to this trial:

**Figure:**
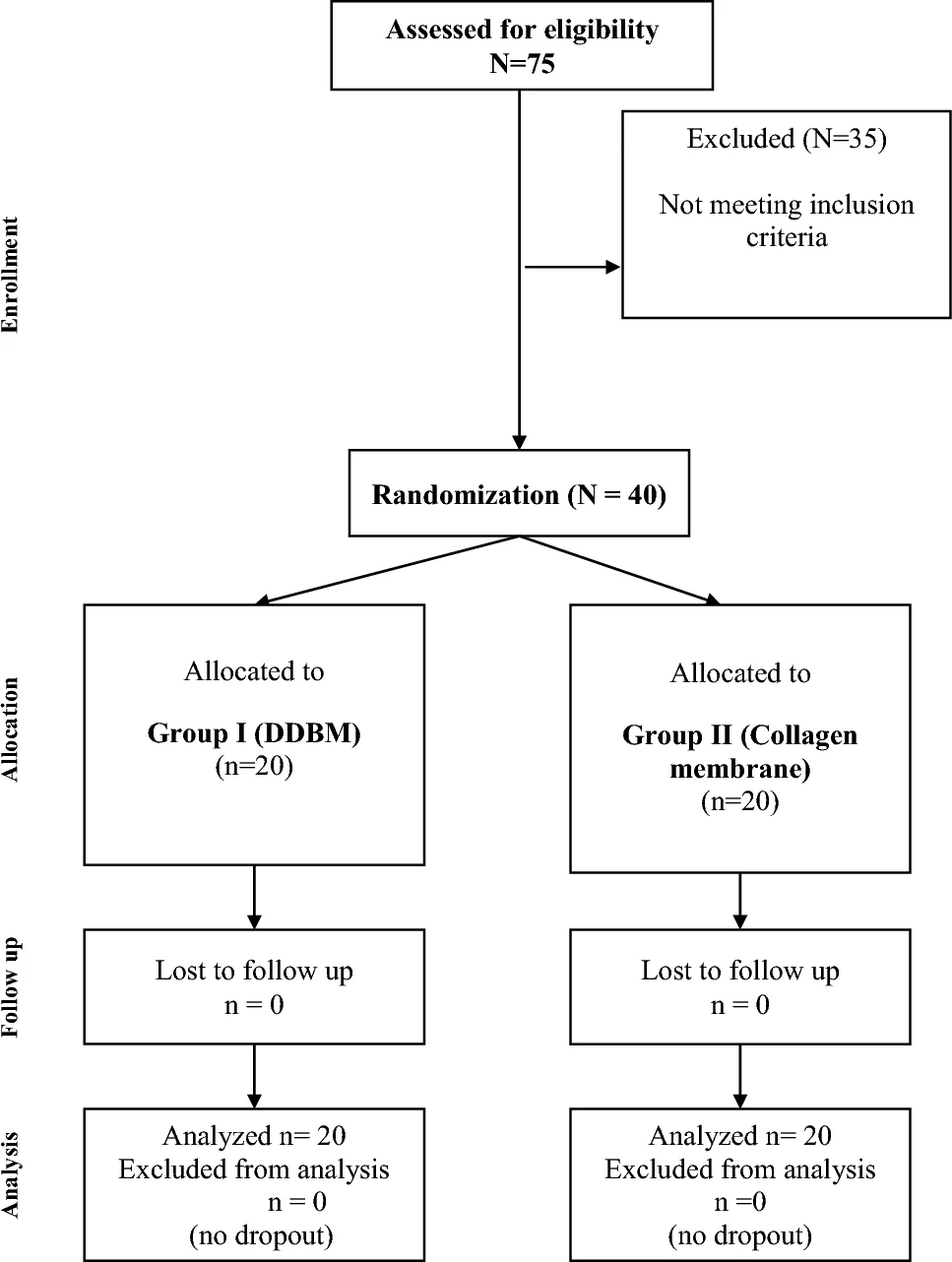

#### Group I

20 immediate implants were inserted after extraction of non-restorable mandibular premolars or molars in conjunction with DDBM from the extracted tooth and xenogeneic bone graft material.

#### Group II

20 immediate implants were inserted after extraction of non-restorable mandibular premolars or molars in conjunction with a collagen membrane and xenogeneic bone graft material.

### Preoperative measurements

**Fig. 1:**
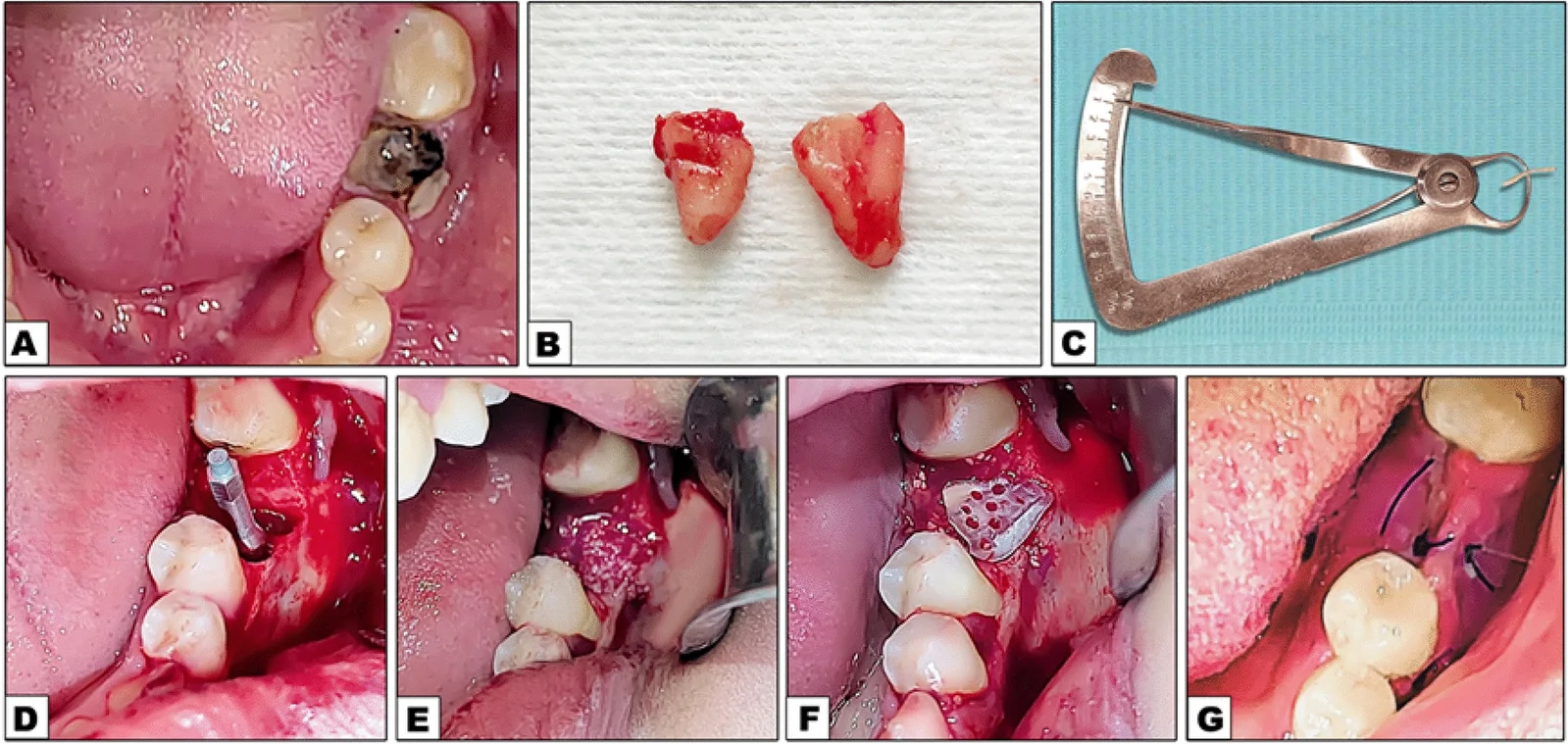
DDBM Group. **A** Preoperative occlusal view of non-restorable lower left 1st molar; **B** Extracted tooth roots; **C** DDBM with thickness 800 µm; **D** Implant placement with smart peg attached to measure primary stability; **E** Xenograft application; **F** DDBM in place; **G** Flap closure and suturing

**Fig. 2:**
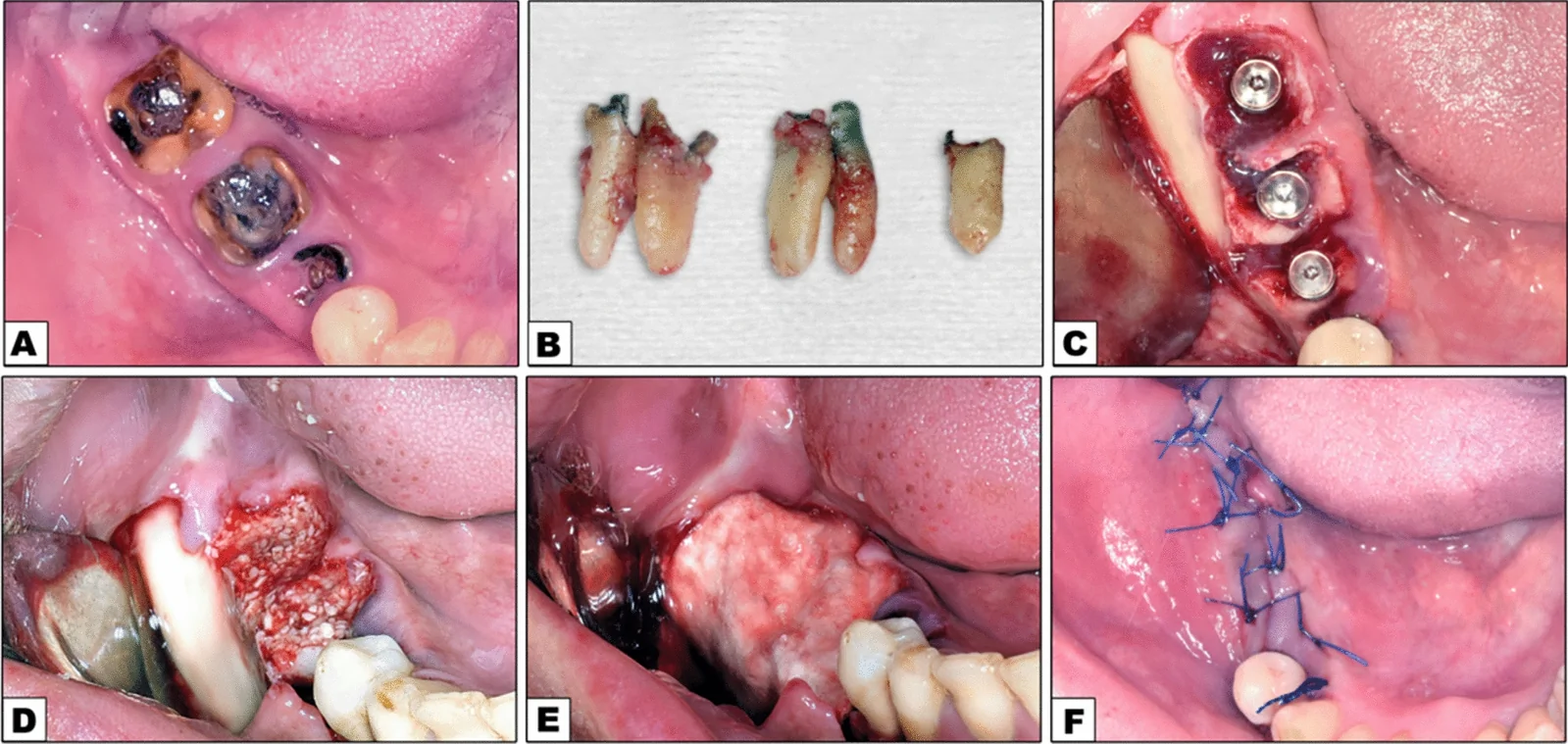
Collagen Membrane Group. **A** Preoperative occlusal view of non-restorable teeth; **B** Extracted teeth roots; **C** Implant in place; **D** Xenograft application; **E** Collagen membrane application; **F** Flap closure and suturing

**Fig. 3:**
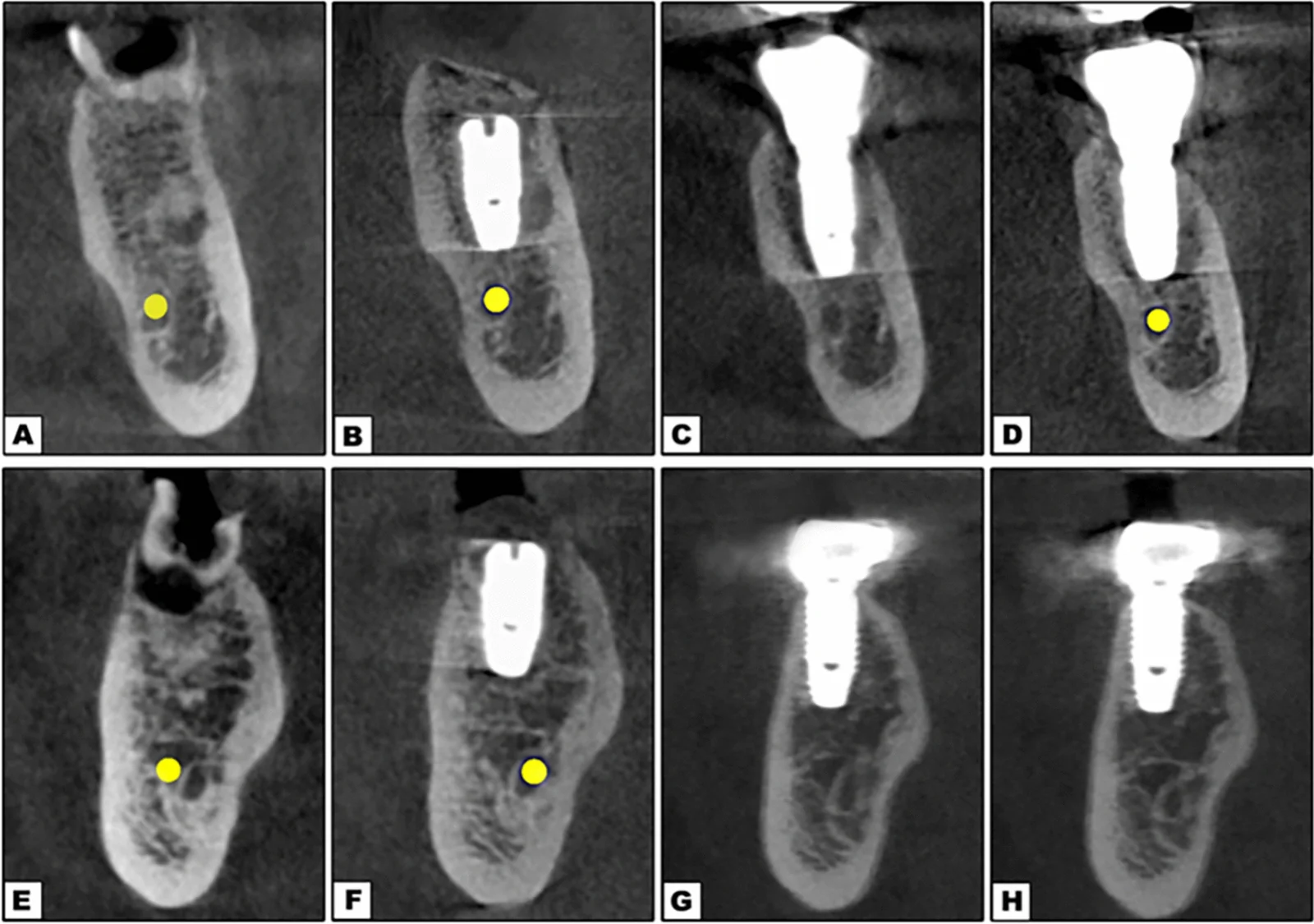
Radiographic Evaluation. DDBM Group **(A–D):** **A** Preoperative; **B** Immediate postoperative; **C** 3 months postoperative (At loading); **D** 9 months postoperative. Collagen membrane Group **(E–H):** **E** Preoperative; **F** Immediate postoperative; **G** 3 months postoperative (At loading); **H** 9 months postoperative

1. Complete dental and medical history were obtained from every patient.
2. Clinical examination was conducted both intra- and extra-orally using palpation and inspection.
3. Study cast were mounted on a semi-adjustable articulator to assess occlusion, crown height space, and teeth inclination.
4. Prior to starting the surgical procedures, a preoperative photo was taken as a baseline document (Figs.1A and 2A).
5. Cone beam computed tomography (CBCT) was obtained to evaluate the surgical site and to properly choose the right implant size (Fig 3A and E).
6. Preoperative medication: Amoxicillin with clavulanic acid 1gm/tab (Augmentin, GlaxoSmithKline, UK) was administered as a prophylactic antibiotic every 12 h for two days before the surgery, and the patients were instructed to use mouthwash containing 0.12% chlorhexidine to rinse their mouth (Orovex, Macro pharmaceuticals, Egypt.) for 30 s immediately before surgery.

### Surgical protocol

- All patients were anesthetized via local infiltration technique (2% mepivacaine with 1: 20,000 levonordefrin) (2% mepivacaine HCL with 1:20,000 levonordefrin. Alexandria, Egypt: Alexandria Co. for Pharmaceuticals and Chemical Industries).
- After making a three-line incision, the mucoperiosteal flap was reflected.
- The tooth or the root was then extracted atraumatically (Figs.1B and 2B).
- Adequate curettage and irrigation of the socket was performed to remove any granulation tissue.
- The manufacturer’s suggested drilling sequence was followed. Always checking the drilling angulation with a paralleling pin.
- Two-piece tapered internal connection titanium implants (IS II Active, Neobiotech®, Seoul, South Korea) with SLA surface were utilized. Implant diameters ranged from 4.0 to 4.5 mm and lengths ranged from 10 to 11.5 mm selected according to preoperative CBCT. They were inserted to engage the bone apical to the socket to obtain sufficient primary stability with an insertion torque of at least 35 Ncm. The implant platform was placed 5 mm apical to the free gingival margin (Fig. 2C).
- A periosteal release incision at the deepest part of the mucoperiosteal flap was done to ensure tension-free closure.
- The gap around the implant and any peri-implant defects were grafted appropriately with xenogeneic bone graft material (OneXeno Graft®, Cortico-cancellous bovine powder, Germany) (Figs.1E and 2D).

### For group I

Preparation of autogenous DDBM [17]:

- The tooth was extracted and submerged in 75% alcohol.
- All the soft tissues and calculus attached to the tooth were removed. Then, the tooth was cleaned, and the remaining periodontal tissue was removed.
- A diamond bur was used to remove cementum and enamel layers. Also, any vital pulp tissue or root canal filling material was removed.
- A micro-fissure bur was used to create additional holes from the dentin surface to the pulp chamber, each with a diameter of 0.2 mm to create macropores to promote vascular invasion and bone formation.
- The tooth was then cut in an occlusal–apical direction, creating a membrane that was 300–800 µm thick (Fig. 1C). Sections that would expose the pulp chamber during the slicing process were thrown away.
- For three minutes, DDBM was immersed in a 10% EDTA solution to partially demineralize the dentin. This allowed the collagen fiber network to be exposed and active growth factors to be released. A buffered saline solution was then used to clean the membrane again.
- DDBM was positioned to carefully cover the alveolar socket up to the height of the buccal and lingual alveolar bones without any additional fixing methods (Fig. 1F).

### For group II

- The sealed package of the collagen membrane (Hypro-Sorb® M, Germany) was opened, then the membrane was trimmed, to ensure sufficient coverage to the grafted site. The membrane’s edges were tucked beneath the flap without buckling or wrinkling (Fig. 2E).

### For both groups

The flap was repositioned and sutured using horizontal mattress and simple interrupted 4/0 polypropylene sutures (Figs.1G and 2F).

### Postoperative care and instructions

- Cold fomentation started immediately postoperatively for the first 24 h.
- To maintain good oral hygiene, the patient was advised to keep using mouthwash that contains chlorohexidine.
- Antibiotic: Amoxicillin with clavulanic acid 1 gm/tab/12 h for 5 days.
- Diclofenac potassium (Cataflam, Alexandria pharmaceutical and chemical industries, Egypt.) 50 mg/tab/8 h for 5 days.
- Sutures were removed 14 days after the surgery.

### 2nd-stage surgery

After three months, the second-stage surgery was performed, during which healing caps were placed for 10 days. The indirect impression technique was then used. Following the evaluation of the crown margins and occlusion, the final crowns were cemented using temporary implant cement.

### Patient’s evaluation

All patients were evaluated clinically and radiographically immediately after surgery, as well as at 3 and 9 months postoperatively.

#### Clinical evaluation

##### Implant stability

Implant stability was assessed immediately after surgery and at 3 and 9 months postoperatively. Resonance frequency analysis (RFA) was used to determine implant stability using an Osstell Mentor device (Osstell, Savadaled, Sweden; Integration Diagnostics). The smart peg type 7 was attached to the dental implant (Fig. 1D). To determine the RFA value, measurements were taken at 90° in four different directions. The outcomes were averaged for every implant and shown as implant stability quotient (ISQ)[18].

##### Landry healing index

All patients were evaluated after 14 days, 1 month, and 3 months after surgery by the Landry healing index [19].

- Score 1: very poor healing.
  - Tissue color: ≥ 50% of gingiva is red.
  - Response to palpation: bleeding.
  - Granulation tissue: present.
  - Incision margin: not epithelialized, with loss of epithelium beyond incision margin.
  - Suppuration: present.
- Score 2: poor healing.
  - Tissue color: ≥ 50% of gingiva red.
  - Response to palpation: bleeding.
  - Granulation tissue: present.
  - Incision margin: not epithelialized, with connective tissue exposed.
- Score 3: good healing.
  - Tissue color: ≥ 25% and < 50% of gingiva red.
  - Response to palpation: no bleeding.
  - Granulation tissue: none.
  - Incision margin: no connective tissue exposed.
- Score 4: very good healing.
  - Tissue color: < 25% of gingiva red.
  - Response to palpation: no bleeding.
  - Granulation tissue: none.
  - Incision margin: no connective tissue exposed.
- Score 5: excellent healing.
  - Tissue color: all tissues pink.
  - Response to palpation: no bleeding.
  - Granulation tissue: none.
  - Incision margin: no connective tissue exposed.

##### Modified sulcus bleeding index (mSBI)

The mSBI measures peri-implant mucosal inflammation around implants 3 and 9 months after surgery [20].

- Score 0: no bleeding when a periodontal probe was passed along the gingival margin adjacent to the implant.
- Score 1: isolated bleeding visible spot.
- Score 2: a confluent red line on the gingival margin was formed by blood.
- Score 3: heavy or profuse bleeding was present.

##### Peri-implant probing depth (PPD)

The depth of the peri-implant sulcus was measured using a manual PCVUNC12PT periodontal probe (Hu-Friedy®, Chicago, IL, USA) with 1-mm graduations up to 12 mm, with light force at 3 and 9 months after surgery. In a line parallel to the implant’s vertical axis, the probe was placed buccally, lingually, mesially, and distally around the implant. The measurements were estimated to the closest 0.5 mm [21].

#### Radiographic evaluation

CBCT was performed immediately after surgery, and after 3 and 9 months. All CBCT scans were carried out at the same radiology center (Planmeca, ProMax® 3D Max, Helsinki, Finland) with identical parameters (89 kVp and 8 mA) For image processing and reconstruction, OnDemand3D was used (Fig. 3).

##### Marginal bone loss

By adjusting the cross section in the middle of the implant, a line was drawn just parallel to the implant, from the crest of the buccal plate of bone to the apical level of the implant. The height was measured in millimeters at the time of surgery and after 3 and 9 months. From the lingual side, the same procedure was carried out again. The baseline value was the bone height measurements taken immediately after implant placement. The difference between the baseline value and the readings obtained 3 and 9 months following surgery was considered MBL [22].

##### Relative bone density

Relative bone density was measured from the bucco-lingual aspect of the cross-sectional view using the Hounsfield unit. For each implant site, six records were measured and averaged. Measurements were taken 1 mm away, parallel to the implant body. From the buccal side, three measurements were made (coronal, middle, and apical thirds), followed by three measurements from the lingual side (in the same manner) [23].

## Statistical analysis

Data were analyzed using SPSS version 27.0. Qualitative data were presented as number and percent. Quantitative data were tested for normality by Shapiro–Wilk test, then described as mean and standard deviation for normally distributed data. Student *t*-test was used to compare between different groups. All P values are two-sided. *P* values ≤ 0.05 were considered significant.

## Results

A total of 35 patients participated in this study, including 20 females (57.1%) and 15 males (42.9%), all seeking replacement of non-restorable single or multiple posterior mandibular teeth. The patients’ ages ranged from 26 to 44 years (Table 1).

**Table 1:** Demographic data among study groups

	Group I *n* = 20	Group II *n* = 20	Test of significance
Age/years (Mean ± SD)	36.70 ± 6.89	36.50 ± 4.64	*t* = 0.108 *P* = 0.915
Sex			
Male	8(44.5%)	7(41.2%)	*P* = 1.0
Female	10(55.5%)	10(58.8%)	

*SD* standard deviation, *P* < 0.05 is statistically significant, analysis was done by student *t*-test for age and fisher exact test for gender

A total of 40 implants were placed to restore 10 s molars (25%), 12 first molars (30%), 6 s premolars (15%), and 12 first premolars (30%) (Table 2). The implants used measured 10 mm or 11.5 mm in length and 4 mm or 4.5 mm in diameter (Table 3).

**Table 2:** Replaced tooth among study groups

Replaced tooth	Group I *n* = 20	Group II *n* = 20	*χ*^2^	*P* value
First premolar	8(40%)	4(20%)	1.90	0.167
Second premolar	2(10%)	4(20%)	0.784	0.378
First molar	4(20%)	8(40%)	1.90	0.167
Second molar	6(30%)	4(20%)	0.533	0.465

*P* < 0.05 is statistically significant, analysis was done by *χ*^2^=Chi-Square test,

**Table 3:** Implant characteristics (length and diameter) among study groups

Implant characters	Group I *n* = 20	Group II *n* = 20	Student *t*-test	*P* value
Implant length (mm)	10.60 ± 0.75	10.6 ± 0.75	0	1.0
Implant diameter (mm)	4.30 ± 0.25	4.30 ± 0.25	0	1.0

*P* < 0.05 is statistically significant, analysis was done by student *t*-test

All implants achieved successful osseointegration with no reported complications, resulting in a 100% success rate throughout the entire follow-up period.

### Clinical evaluation

#### Implant stability

There were no statistically significant differences between the two groups at the time of implant insertion, or after 3 and 9 months (*P* = 0.866, 0.546, and 0.611, respectively). (Table 4).

**Table 4:** Implant stability

	Time of assessment	Group I *n* = 20	Group II *n* = 20	Test of significance
Implant stability	Implant insertion (*T*_0_)	70.5 ± 2.98	70.7 ± 4.33	*t* = 0.170 *P* = 0.866
	After 3 months (*T*_3_)	75.10 ± 3.02	75.80 ± 4.15	*t* = 0.610 *P* = 0.546
	After 9 months (*T*_9_)	77.5 ± 3.08	78.1 ± 4.22	*t* = 0.514 *P* = 0.611
Follow up comparison#	*T*_0_ − *T*_3m_	*P*1 < 0.001*	*P*1 < 0.001*	
	*T*_0_ − *T*_9m_	*P*2 < 0.001*	*P*2 < 0.001*	
	*T*_3m_ − *T*_9m_	*P*3 < 0.001*	*P*3 < 0.001*	

*t*=Student *t*-test, *statistically significant, #Paired *t*-test

#### Landry healing index

There were no statistically significant differences between the two groups at 14 days, 1 month, and 3 months (*P* = 0.232, 0.813, and 0.776, respectively). (Table 5).

**Table 5:** Landry healing index

Time of assessment	Landary healing index	Group I *n* = 20	Group II *n* = 20	Test of significance
T _14D_	Score 1 Score 2 Score 3 Score 4	6(30) 4(20) 6(30) 4(20)	2(10) 8(40) 8(40) 2(10)	*χ*^2^ = 4.28 *P* = 0.232
T_1m_	Score 2 Score 3 Score 4 Score 5	4(20) 6(30) 8(40) 2(10)	2(10) 8(40) 8(40) 2(10)	*χ*^2^ = 0.952 *P* = 0.813
T_3m_	Score 3 Score 4 Score 5	4(20) 8(40) 8(40)	4(20) 10(50) 6(30)	*χ*^2^ = 0.508 *P* = 0.776
Follow up comparison#	*T*_14D_** − ***T*_1m_	*P* = 0.001*	*P* = 0.001*	
	*T*_14D_** − ***T*_**3**m_	*P* = 0.001*	*P* = 0.001*	
	*T*_**1m**_** − ***T*_**3**m_	*P* = 0.001*	*P* = 0.001*	

*χ*^2^ = Chi-Square test, *statistically significant, #Marginal Homogeneity test

#### Modified sulcus bleeding index

There were no statistically significant differences between the two groups after 3 and 9 months (*P* = 0.81 and 0.79, respectively). (Table 6).

**Table 6:** Modified Sulcus Bleeding Index (mSBI)

	Time of assessment	Group I *n* = 20	Group II *n* = 20	Test of significance
Modified Sulcus Bleeding Index (mSBI)	After 3 months (T3)	1.85 ± 0.42	1.88 ± 0.40	*t* = 0.23 *P* = 0.81
	After 9 months (T9)	1.88 ± 0.21	1.90 ± 0.26	*t* = 0.26 *P* = 0.79
Follow up comparison#	*T*3 − *T*9m	*P* = 0.77	*P* = 0.85	

*t* =Student *t*-test, *statistically significant, #Paired *t*-test

#### Peri-implant pocket depth

No statistically significant differences were found between the two groups after 3 and 9 months (*P* = 0.963 and 1, respectively). (Table 7).

**Table 7:** Peri-implant pocket depth

	Time of assessment	Group I *n* = 20	Group II *n* = 20	Test of significance
Peri-implant pocket depth	3 months (*T*_3_)	1.88 ± 0.72	1.89 ± 0.66	*t* = 0.046 *P* = 0.963
	9 months (*T*_9_)	2.45 ± 0.64	2.45 ± 0.54	*t* = 0.0 *P* = 1.0
Follow up comparison#	*T*_3m_ − *T*_9m_	*P* < 0.001*	*P* < 0.001*	

*t*=Student *t*-test, *statistically significant, #Paired *t*-test

### Radiographic evaluation

#### Marginal bone loss

Regarding buccal MBL, no statistically significant differences were found between the two groups after 3 and 9 months (*P* = 0.42 and 0.81, respectively). (Table 8).

**Table 8:** Marginal bone Loss (MBL)

Marginal bone Loss	Time of assessment	Group I *n* = 20	Group II *n* = 20	Test of significance
**Buccal**	After 3 months (T_3_)	0.690 ± 0.32	0.616 ± 0.25	*t* = 0.81 *P* = 0.42
	After 9 months (T9)	1.42 ± 0.307	1.40 ± 0.228	*t* = 0.23 *P* = 0.81
		*P* < 0.001*	*P* < 0.001*	
**Lingual**	After 3 months (T_3_)	0.733 ± 0.203	0.692 ± 0.15	*t* = 0.72 *P* = 0.47
	After 9 months (T9)	1.31 ± 0.12	1.29 ± 0.32	*t* = 0.26 *P* = 0.79
Follow up comparison#		*P* < 0.001*	*P* < 0.001*	

*t*=Student *t*-test, *statistically significant, #Paired *t*-test

Similarly, for lingual MBL, no statistically significant differences were observed between the two groups after 3 and 9 months (*P* = 0.47 and 0.79, respectively). (Table 8).

#### Relative bone density

When comparing both groups, no statistically significant differences were found immediately after surgery and at 3 and 9 months (*P* = 0.882, 0.660, and 0.858, respectively) (Table 9).

**Table 9:** Relative bone density

	Time of assessment	Group I *n* = 20	Group II *n* = 20	Test of significance
Relative bone density	Implant insertionT_0_	613.62 ± 187.95	622.18 ± 174.46	*t* = 0.149 *P* = 0.882
	After 3 months (*T*_3_)	822.05 ± 246.92	854.07 ± 208.78	*t* = 0.443 *P* = 0.660
	After 9 months (*T*_9_)	993.32 ± 178.89	1003.35 ± 173.53	*t* = 0.180 *P* = 0.858
Follow up comparison#	*T*_0_−*T*_3m_	*P* < 0.001*	*P* < 0.001*	
	*T*_0_−*T*_9m_	*P* < 0.001*	*P* < 0.001*	
	*T*_3m_−*T*_9m_	*P* < 0.001*	*P* < 0.001*	

*t*=Student *t*-test, *statistically significant, #Paired* t*-test

## Discussion

Immediate implant placement is a popular approach for replacing non-restorable teeth because it preserves the alveolar ridge, prevents adjacent teeth from drifting into the edentulous area, and reduces overall treatment time [24]. However, its drawbacks include the mismatch between implant size and the extraction socket, as well as the gap that often occurs coronally between the implant and the socket wall due to the large socket left after tooth removal [24]. This area resembles a circumferential vertical defect and may provide a space for soft tissue ingrowth. Therefore, GBR is often required during immediate implant placement to preserve both soft and hard tissue contours [25].

In GBR, membranes are used to prevent the migration of fibrogenic cells while allowing osteoprogenitor cells to populate the defect area, as osteoprogenitor cells proliferate more slowly than fibrogenic cells. Consequently, several studies have been conducted to enhance bone regeneration beneath the membrane [26, 27].

The implants used in this study demonstrated a 100% survival rate. This success can be attributed to careful patient selection, strict patient compliance with oral hygiene instructions throughout the follow-up period, and the use of appropriate surgical techniques.

The rationale of this study was to evaluate the clinical and radiographic outcomes of autogenous DDBM compared with a collagen membrane for GBR in conjunction with immediate implant placement in the posterior mandible.

Several studies have used dentin particulate as a grafting material for socket preservation or for filling bone defects [28, 29]. Other studies have utilized tooth shells for alveolar ridge augmentation [30, 31]. However, only one case report has described the use of a DDBM for GBR in conjunction with early implant placement, four weeks after extraction [17].

In the present study, the DDBM combined with xenograft and transformed into a uniform, hard tissue surrounding the implant. DDBM has the ability to protect the bone graft. Additionally, the DDBM’s ability to quickly remodel using endogenous bone morphogenic protein (BMP) from dentin allows for the bone formation [17]. The enlargement of dentinal tubules resulting from the demineralization process may enhance the release of endogenous growth factors from the dentin matrix, thereby promoting osteoinductivity through the phenotypic conversion of fibroblasts into osteoblasts [15]. Following demineralization, the remaining mineral component in the DDBM forms a natural Type I collagen membrane with a thickness ranging from 300 to 800 µm, which is biomimetic to cortical bone [32]. The inherent micropores (dentinal tubules) in the DDBM range from 3 to 5 µm in diameter, while the macropores measure between 0.2- and 0.3-mm. Regarding membrane porosity, which plays a critical role in both soft and hard tissue regeneration, larger pore sizes have been shown to enhance cellular and nutrient infiltration, as well as facilitate the diffusion of cells, growth factors, and nutrient-rich blood supply [7].

The collagen membrane was used in this study because the cells recruited into it can stimulate the production of bone morphogenetic proteins (BMPs) and other osteogenic factors. Consequently, the membrane served a role similar to that of the periosteum, functioning not only as a passive barrier but also as a biologically active component in bone regeneration [17].

No extra fixing technique was used for both groups. In DDBM group, the intimate adaptation of the flap guaranteed the stability of the dentin membrane, which has enough rigidity to preserve space without fixation. In collagen membrane group, in order to provide mechanical stability and prevent displacement, the collagen membrane was carefully adapted to the grafted site, with its edges tucked beneath the mucoperiosteal flap without buckling or wrinkling.

Primary flap closure, achieved through periosteal releasing incisions to achieve tension-free primary closure, is an essential step to prevent infection and epithelial downgrowth at the implant site [33]. In our study no membrane exposure cases were observed during the follow-up period suggests that the surgical technique provided complete soft tissue coverage. However, it is noteworthy that a previous study using an autogenous tooth bone graft block has demonstrated the ability to accomplish secondary healing if the graft gets exposed, such as in a wound dehiscence condition [32].

Regarding the implant stability, no statistically significant differences were found at the time of implant insertion or after 3 and 9 months (*P* > 0.05) when comparing the two groups. However, within each group, a statistically significant increase in implant stability (ISQ values) was observed throughout the entire follow-up period.

This can be explained by the intimate contact between the implant and the apical bone, which provides both groups with excellent primary stability during insertion [34]. Both groups demonstrated improved stability after 3 months, attributed to new bone deposition and osseointegration around the implant [35]. The further improvement in stability after 9 months may be attributed to biomechanical stimulation from functional occlusal loading, which promotes remodeling and reorganization of bone trabeculae at the bone–implant interface. This enhances load transfer efficiency and optimizes stress distribution within the supporting bone [36].

This finding is in line with Witoonkitvanich et al. [37], who clarified that the stability of immediate implants primarily results from the mechanical contact between the implant and the apical bone. At this stage, there is no organic network connecting the implant to the surrounding bone, a condition referred to as primary stability. Subsequently, bone formation and remodeling lead to osseointegration, which establishes secondary or biological stability.

Similarly, Berglundh et al. [38] reported that implant ISQ values tend to increase during functional loading and follow-up periods. This can be attributed to the process of bone adaptation to mechanical stress, during which immature woven bone transforms into well-organized lamellar bone.

The Landry healing index was used to evaluate wound healing. Analysis of changes over time within each group revealed that both groups demonstrated progressive improvement in Landry index scores from 14 days to 3 months. This enhancement can be attributed to the use of mouthwash containing 0.12% chlorhexidine, which has been shown to significantly reduce gingivitis, bleeding, and plaque compared to other mouthwashes, thereby promoting wound healing [39]. When comparing both groups, no statistically significant differences were observed at 14 days, 1 month, or 3 months (*P* > 0.05).

These findings are consistent with those reported by Li et al. [30], Awad et al. [31], and Elsheikh et al. [40]. No complications, such as delayed healing, wound dehiscence, or implant failure, were encountered. The use of autogenous DDBM proved to be safe, as it is derived from autogenous tissue and is not expected to elicit any immunological or infectious response [11].

This study evaluated the peri-implant mucosa at 3 and 9 months postoperatively using the mSBI. When comparing the two groups, no statistically significant differences were observed (*P* > 0.05). Consistent with our findings, Meijer and Raghoebar [41] emphasized that strict patient compliance with postoperative oral hygiene instructions contributes to maintaining excellent peri-implant soft tissue health.

PPD measurements were less than 2.5 mm in both groups at all follow-up intervals. In agreement with our findings, Monje and Salvi [42] stated that a distance of < 6 mm between the mucosal margin and the base of the pocket indicates a healthy peri-implant condition, while ≥ 6 mm is suggestive of peri-implantitis. Likewise, Adell et al. [43] reported that implants with probing depths less than 3 mm are considered healthy.

Regarding MBL, both groups showed minimal buccal and lingual MBL. Our study was in line with Bungthong et al. [44], who reported 0.69 mm buccal MBL and 0.39 mm lingual MBL after 6 months. Also, our study was consistent with Tallarico et al. [45] and Checchi et al. [46], who had bone loss of 0.23 mm and 0.68 mm respectively, from initial loading to 1-year evaluation.

Regarding relative bone density, intragroup pairwise comparisons revealed a significant increase in bone density over time, with the highest values observed after 9 months (*P* < 0.001). When comparing both groups, no statistically significant differences were found immediately postoperatively or after 3 and 9 months (*P* > 0.05). Our study was in line with Issa et al. [35], who observed that bone density values in the apical, body, and neck regions of the implant increased 3 months after insertion and continued to rise 2 months after the prosthesis was placed.

## Limitations

The nine-month follow-up period provides valuable short-term insights, and the results are noteworthy; however, long-term studies are still required to validate the stability of regenerated bone and peri-implant soft tissue parameters over time.

## Conclusion

Within the limitations of this study, DDBM is totally biocompatible because it is an autogenous material, which eliminates the possibility of disease transmission while enabling the clinical recycling of extracted teeth. DDBM demonstrated effectiveness in achieving successful GBR. Therefore, in implant dentistry, DDBM can be regarded as a novel, natural collagen membrane that mimics the osteogenetic role of the periosteum and functions as an osteoinductive membrane with favorable mechanical and clot stability. Although, DDBM requires additional chairside preparation time, its autogenous origin, inherent biological activity, and potential cost-effectiveness, DDBM may represent a promising alternative to the collagen membrane in GBR procedures.

## Data Availability

The corresponding author can provide the data sets utilized and/or analyzed for this study upon reasonable request.

## Abbreviations

GBR: Guided bone regeneration
DDM: Demineralized dentin matrix
DBBM: Deproteinized bovine bone mineral
DDBM: Dentine-derived barrier membrane
CBCT: Cone beam Computed tomography
RFA: Resonance frequency analysis
ISQ: Implant stability quotient
mSBI: Modified sulcus bleeding index
PPD: Peri-implant probing depth
MBL: Marginal bone loss
HU: Hounsfield units
BMP: Bone morphogenic protein

## References

[1] Dhami B, Shrestha P, Gupta S, Pandey N. Immediate implant placement: current concepts. J Nepal Soc Periodontol Oral Implantol. 2019;3(1):18–24. 10.3126/jnspoi.v3i1.24823.

[2] Chappuis V, Araújo MG, Buser D. Clinical relevance of dimensional bone and soft tissue alterations post‐extraction in esthetic sites. Periodontol 2000. 2017;73(1):73–83. 10.1111/prd.12167. 28000281

[3] de Araújo Nobre M, Antunes C, Lopes A, Ferro A, Nunes M, Gouveia M, et al. Partial implant rehabilitations in the posterior regions of the jaws supported by short dental implants (7.0 mm): a 7-year clinical and 5-year radiographical prospective study. J Clin Med. 2024;13(6):1549. 10.3390/jcm13061549. PMC10971251 38541775

[4] Franke T, Korzinskas T. Guided bone regeneration in the posterior mandible using a resorbable metal magnesium membrane and fixation screws: a case report. Case Rep Dent. 2024;1:2659893. 10.1155/crid/2659893. PMC11661873 39713245

[5] Patil S, Bhandi S, Bakri MMH, Albar DH, Alzahrani KJ, Al-Ghamdi MS, et al. Evaluation of efficacy of non-resorbable membranes compared to resorbable membranes in patients undergoing guided bone regeneration. Heliyon. 2023;9(3):e13488. 10.1016/j.heliyon.2023.e13488. PMC10024103 36942236

[6] Bianchi S, Bernardi S, Simeone D, Torge D, Macchiarelli G, Marchetti E. Proliferation and morphological assessment of human periodontal ligament fibroblast towards bovine pericardium membranes: an *in vitro* study. Materials. 2022;15(23):8284. 10.3390/ma15238284. PMC9740786 36499781

[7] Elgali I, Omar O, Dahlin C, Thomsen P. Guided bone regeneration: materials and biological mechanisms revisited. Eur J Oral Sci. 2017;125(5):315–37. 10.1111/eos.12364. PMC5601292 28833567

[8] Pellegrini G, Pagni G, Rasperini G. Surgical approaches based on biological objectives: GTR versus GBR techniques. Int J Dent. 2013;2013:521547. 10.1155/2013/521547. PMC3697289 23843792

[9] Bernardi S, Marchetti E, Torge D, Simeone D, Macchiarelli G, Bianchi S. Ultrastructural assessment of human periodontal ligament fibroblast interaction with bovine pericardium membranes: an in vitro study. Histol Histopathol. 2025;40(8):1185–94. 10.14670/HH-18-860. 39723586

[10] Sapoznikov L, Humphrey M. Progress in dentin-derived bone graft materials: a new xenogeneic dentin-derived material with retained organic component allows for broader and easier application. Cells. 2024;13(21):1806. 10.3390/cells13211806. PMC11544873 39513913

[11] Murata M, Okubo N, Shakya M, Kabir MA, Yokozeki K, Zhu B, et al. Dentin materials as biological scaffolds for tissue engineering. Biomater Tissue Reconstr or Regen. 2019. 10.5772/intechopen.85452.

[12] Um IW, Ku JK, Lee BK, Yun PY, Lee JK, Nam JH. Postulated release profile of recombinant human bone morphogenetic protein-2 (rhBMP-2) from demineralized dentin matrix. J Korean Assoc Oral Maxillofac Surg. 2019;45(3):123–8. 10.5125/jkaoms.2019.45.3.123. PMC6620303 31334099

[13] Bianchi S, Bernardi S, Mattei A, Cristiano L, Mancini L, Torge D, et al. Morphological and biological evaluations of human periodontal ligament fibroblasts in contact with different bovine bone grafts treated with low-temperature deproteinisation protocol. Int J Mol Sci. 2022;23(9):5273. 10.3390/ijms23095273. PMC9101062 35563664

[14] Korsch M. Tooth shell technique: a proof of concept with the use of autogenous dentin block grafts. Aust Dent J. 2021;66(2):159–68. 10.1111/adj.12814. 33370452

[15] Um IW, Ku JK, Kim YK, Lee BK, Leem DH. Histological review of demineralized dentin matrix as a carrier of rhBMP-2. Tissue Eng Part B Rev. 2020;26(3):284–93. 10.1089/ten.teb.2019.0291. PMC7310192 31928139

[16] Mizar MA, Hassan KS, Mohammed AK, Mwafy IM. Platelet rich fibrin versus collagen membrane combined with beta tri calcium phosphate/collagen for treatment of dehiscence around immediately placed implants clinical and radiographic comparative study. Al-Azhar Assiut Dental J. 2021;4(2):189–96. 10.21608/aadj.2021.208221.

[17] Ku JK, Um IW, Jun MK, Kim I. Dentin-derived-barrier membrane in guided bone regeneration: a case report. Materials. 2021;14(9):2166. 10.3390/ma14092166. PMC8123078 33922832

[18] Meredith N. Assessment of implant stability as a prognostic determinant. Int J Prosthodont. 1998;11(5):491–501. 9922740

[19] Hamzani Y, Chaushu G. Evaluation of early wound healing scales/indexes in oral surgery: a literature review. Clin Implant Dent Relat Res. 2018;20(6):1030–5. 10.1111/cid.12680. 30324746

[20] Mombelli A, van Oosten MA, Schurch E Jr, Land NP. The microbiota associated with successful or failing osseointegrated titanium implants. Oral Microbiol Immunol. 1987;2(4):145–51. 10.1111/j.1399-302x.1987.tb00298.x. 3507627

[21] Morgulis JR. Indices of periodontal disease. Periodontal Abstr. 1975;23(1):13–20. 146849

[22] Mounir M, Beheiri G, El-Beialy W. Assessment of marginal bone loss using full thickness versus partial thickness flaps for alveolar ridge splitting and immediate implant placement in the anterior maxilla. Int J Oral Maxillofac Surg. 2014;43(11):1373–80. 10.1016/j.ijom.2014.05.021. 24973295

[23] Haggag M, Said W, Attia A, Tawfik M. Evaluation of bone density after bone condensation around immediate loaded dental implants using different techniques. J Am Sci. 2020;16(7):33–42. 10.7537/marsjas160720.06.

[24] Kabi S, Kar R, Samal D, Deepak KC, Kar IB, Mishra N. Immediate dental implant placement with or without autogenous bone graft: a comparative study. Natl J Maxillofac Surg. 2020;11(1):46–52. 10.4103/njms.NJMS_59_19. PMC7518474 33041576

[25] Cirmeni M, Fedele O, Giammarinaro E, Marconcini S, Covani U, Caso G. Immediate implant and socket preservation using sticky bone and leukocyte-platelet-rich fibrin in the anterior maxilla: a 3-year case report. Clin Adv Periodontics. 2023;13(3):144–8. 10.1002/cap.10202. 35581730

[26] Li W, Ma G, Brazile B, Li N, Dai W, Butler JR, et al. Investigating the potential of amnion-based scaffolds as a barrier membrane for guided bone regeneration. Langmuir. 2015;31(31):8642–53. 10.1021/acs.langmuir.5b02362. 26158559

[27] Um IW, Ku JK, Kim YM, Yun PY, Chang NH, Kim YK, et al. Allogeneic demineralized dentin matrix graft for guided bone regeneration in dental implants. Appl Sci. 2020;10(13):4661–74. 10.3390/app10134686.

[28] Cervera-Maillo JM, Morales-Schwarz D, Morales-Melendez H, Mahesh L, Calvo-Guirado JL. Autologous tooth dentin graft: a retrospective study in humans. Medicina (B Aires). 2021;58(1):56. 10.3390/medicina58010056. PMC8778028 35056364

[29] Fathy AM, Abd-ElAkher MH, Elfeky AA. Effect of using mineralized dentin particulate grafted for socket preservation. Al-Azhar J Dent Sci. 2019;22(3):211–5. 10.21608/ajdsm.2019.108445.

[30] Li S, Gao M, Zhou M, Zhu Y. Bone augmentation with autologous tooth shell in the esthetic zone for dental implant restoration: a pilot study. Int J Implant Dent. 2021;7(1):108. 10.1186/s40729-021-00389-w. PMC8575770 34748111

[31] Awad KAI, Tawik MAM, Hussein MM, El-Farag SAA, Sameaa SESA. Tooth shell versus bone shell technique for horizontal maxillary alveolar ridge augmentation. BMC Oral Health. 2025;25(1):642. 10.1186/s12903-025-05940-4. PMC12032675 40281533

[32] Kim YK, Kim SG, Um IW, Kim KW. Bone grafts using autogenous tooth blocks: a case series. Implant Dent. 2013;22(6):584–93. 10.1097/ID.0000000000000011. 24225779

[33] Elsheikh HAE, Abdelsameaa SE, Elbahnasi AA, Abdel-Rahman FH. Comparison between platelet rich fibrin as space filling material versus xenograft and alloplastic bone grafting materials in immediate implant placement: a randomized clinical trial. BMC Oral Health. 2023;23(1):977. 10.1186/s12903-023-03678-5. PMC10704731 38066454

[34] Lee W. Immediate implant placement in fresh extraction sockets. J Korean Assoc Oral Maxillofac Surg. 2021;47(1):57–61. 10.5125/jkaoms.2021.47.1.57. PMC7925164 33632979

[35] Issa NSH, Othman TA, Sleman BM. A comparative radiographic study of bone density changes around titanium implants in the posterior mandible, preoperative, and postoperative. Ann Med Surg (Lond). 2024;86(6):3216–21. 10.1097/MS9.0000000000002142. PMC11152818 38846880

[36] Brizuela-Velasco A, Chávarri-Prado D. The functional loading of implants increases their stability: a retrospective clinical study. Clin Implant Dent Relat Res. 2019;21(1):122–9. 10.1111/cid.12702. 30548792

[37] Witoonkitvanich P, Amornsettachai P, Panyayong W, Rokaya D, Vongsirichat N, Suphangul S. Comparison of the stability of immediate dental implant placement in fresh molar extraction sockets in the maxilla and mandible: a controlled, prospective, non-randomized clinical trial. Int J Oral Maxillofac Surg. 2025;54(4):365–73. 10.1016/j.ijom.2024.12.011. 39794221

[38] Berglundh T, Abrahamsson I, Lang NP, Lindhe J. De novo alveolar bone formation adjacent to endosseous implants: a model study in the dog. Clin Oral Implants Res. 2003;14(3):251–62. 10.1034/j.1600-0501.2003.00972.x. 12755774

[39] Ferrés-Amat E, Al Madhoun A, Ferrés-Amat E, Carrió N, Barajas M, Al-Madhoun AS, et al. Comparison of 0.12% chlorhexidine and a new bone bioactive liquid, BBL, in mouthwash for oral wound healing: a randomized, double blind clinical human trial. J Pers Med. 2022;12(10):1725. 10.3390/jpm12101725. PMC9605239 36294864

[40] Elsheikh S, Noureldin M. Evaluation of bone augmentation using autologous tooth shell in the esthetic zone of narrow maxilla (a clinical study). Alexandria Dent J. 2025;50(1):110–6. 10.21608/adjalexu.2024.240808.1424.

[41] Meijer HJA, Raghoebar GM. Immediate implant placement in molar extraction sites: a 1-year prospective case series pilot study. Int J Implant Dent. 2020;6(1):3. 10.1186/s40729-019-0201-7. PMC7013013 32048081

[42] Monje A, Salvi GE. Diagnostic methods/parameters to monitor peri‐implant conditions. Periodontol 2000. 2024;95(1):20–39. 10.1111/prd.12584. 38923148

[43] Adell R, Lekholm U, Rockler B, Brånemark PI. A 15-year study of osseointegrated implants in the treatment of the edentulous jaw. Int J Oral Surg. 1981;10(6):387–416. 10.1016/S0300-9785(81)80077-4. 6809663

[44] Bungthong W, Amornsettachai P, Luangchana P, Chuenjitkuntaworn B, Suphangul S. Bone dimensional change following immediate implant placement in posterior teeth with CBCT: a 6-month prospective clinical study. Molecules. 2022;27(3):608. 10.3390/molecules27030608. PMC8838578 35163869

[45] Tallarico M, Xhanari E, Pisano M, Gatti F, Meloni SM. Molar replacement with 7 mm-wide diameter implants: to place the implant immediately or to wait 4 months after socket preservation? 1 year after loading results from a randomised controlled trial. Eur J Oral Implant. 2017;10(2):169–78. 28555207

[46] Checchi V, Felice P, Zucchelli G, Barausse C, Piattelli M, Pistilli R, et al. Wide diameter immediate post-extractive implants vs delayed placement of normal-diameter implants in preserved sockets in the molar region: 1-year post-loading outcome of a randomised controlled trial. Eur J Oral Implantol. 2017;10(3):263–78. 28944355

